# Traditional management of ear, nose and throat (ENT) diseases in Central Kenya

**DOI:** 10.1186/1746-4269-2-54

**Published:** 2006-12-27

**Authors:** Grace N Njoroge, Rainer W Bussmann

**Affiliations:** 1 Jomo Kenyatta University of Agriculture and Technology, Botany Department P.O. Box 62000, Nairobi, Kenya; 2 University of Hawaii, Botany, 3860 Manoa Rd, Honolulu HI 96822, USA

## Abstract

Diseases of ear, nose and throat (ENT) often have serious consequences including hearing impairment, and emotional strain that lower the quality of life of patients. In Kenya, upper respiratory infections are among the most common infections encountered in outpatient facilities. Some of these infections are becoming difficult to control because some of the causing microorganisms have acquired antibiotic resistance and hence the need to develop new drugs with higher efficacy. Ethnobotanical studies have now been found to be instrumental in improving chances of discovering plants with antimicrobial activity in new drug development. In Kenya the majority of local people are turning to herbal remedies for primary health care needs. In most cases the sources of these remedies are undocumented and the knowledge about them passed orally form generation to generation, hence under threat of disappearing with current rates of modernisation.

This study explored the traditional remedies used in managing various ENT diseases in seven districts of the Central Province of Kenya. The most common ENT conditions managed using traditional therapies include: common cold, cough, tonsillitis, otitis-media, chest pains and asthma. The results indicate that 67 species belonging to 36 plant families were utilized in this region. These plants were of varying habits; herbs (37.3%), shrubs (34.4%), trees (25.4%) as well as some grasses and sedges (3%). The traditional preparations were found to be made mainly from leaves (49%), roots (20.5%) and barks (12.5%). For each of the ENT conditions multiple species are utilized mainly as individual preparations but occasionally as polyherbal concoctions. In the case of common cold for example, 30 different species are used. Plants reported in this survey are important candidates for antimicrobial tests against ENT disease causing micro-organisms, especially those with antibiotic resistance.

## Background

Diseases of the ear, nose and throat (ENT) affect the functioning of adults as well as children, often with significant impairment of the daily life of affected patients [1]. It has been envisaged that with increase in global population, infections remain the most important causes of disease, with upper respiratory infections causing hearing loss and learning disability particularly in children [2]. Ear infections such as chronic otitis-media have serious consequences in developing countries, such as retarded language development and progress in school among children [3,4]. Otitis-media, which is now known to be the most common childhood infection, leads annually to the death of over 50,000 children under 5 years [5]. In other cases nasal conditions may be distressing, as in the case of nasal myiasis/maggots in the nose [6].

In most countries in the developing world the number of otolaryngologists is negligible, while the problem is complicated by the fact that there is no training for public health otolaryngology and other ENT-related otolaryngology personnel [2,4]. This lack of trained personnel is of particular concern in African countries because the prevalence rates of some of the ENT disease such as chronic otitis-media is as high as 65% [7]. This problem as well as increased costs of conventional medicine has caused local people in Kenya and in other developing countries to seek treatment from traditional therapies.

Diseases of the ear, nose and throat can be caused by a variety of microorganisms. Rhinoviruses are the leading cause for common cold in all age groups for example, while enteroviruses are frequently associated [8]. Acute phryngitis/nosilitis is mainly associated with respiratory viruses, although bacteria, especially *Streptococus *spp. are found in some patients [9]. It has been shown that the nose is the main ecological niche where some of the drug resistant microorganisms like *Staphylococcus aureus *reside [10]. Although acute otitis-media is caused by bacteria the leading one being *Streptococcus pneumoniae *[11], viral infection are a predisposing factor for its development [8,12]. In communities that use antibiotics, *Streptococcus pneumoniae *rapidly acquire antibiotics resistance, often complicating this disease burden [11].

Tuberculosis is regarded as the oldest disease in man, affecting almost every organ of the body and causing about 2 million deaths annually [13]. Cases of tuberculosis are on the increase globally, with HIV/AIDS leading to re-emergence of extra pulmonary presentations [14]. While initial tuberculosis infections occur in the lungs, forms that occur for example in tonsils and the ear are now known to occur particularly in regions such as Africa where the disease is endemic [15].

Although antibiotics have contributed to the control of ENT infections, their over-use and misuse is now seen to cause an increase in antibiotic resistance [16]. Some of the chronic sinus ENT diseases resistant to current antibiotics include chronic middle ear infections, chronic sinus diseases, chronic coughs and recurrent pharyngo-tonsillitis [17]. Air pollution has been on the increase and is now known to directly affect the nose and the larynx causing inflammation, irritations and eventually infection. Even the ear is affected when the pollutants enter the mucosa of the tuba, causing impairment of the middle ear [18]. With increasing resistance of microorganisms associated with ENT infections and increasing environmental pollution, alternative sources for new drugs are necessary. These might be obtained from plants used in traditional medicines to control or treat these diseases. People in Kenya are now turning to the use of traditional herbal medicines so as to meet their primary healthcare needs [19].

In Kenya diseases of the respiratory tract are among the most common illnesses in outpatient clinics [20]. These diseases, combined with malaria, account for half the diseases reported in outpatient facilities [21]. Since some of the ENT disease causing microorganisms have become resistant to current antibiotics, there is need to investigate means of developing new, efficacious drugs.

Studies have now shown that ethobotanically-derived phytochemicals have greater activity than compounds derived from random screening and therefore a greater potential for products developed [22,23]. One study for example, has shown that 86% of plants species reported in Samoan pharmaceutical ethnopharmacopoeia showed pharmacological activity [24]. Further, many drugs in clinical use today were discovered from the way plants were used in traditional communities. Examples include quinine which, was discovered from the way traditional communities in S. America especially Peru, Columbia and Bolivia used plant species of the genus *Cinchona *in managing fevers [25]. Digitoxin on the other hand is a popular heart tonic obtained from *Digitalis purpurea*, a plant that was in use as heart tonic in traditional communities in Europe. Taxol is a modern therapy for ovarian cancer obtained from *Taxus brevifolia *which was a traditional medicinal plant in British Columbia [26].

This study investigated the diversity of traditional medicines used for the management of ear, nose and throat infections among the Kikuyus in Central Kenya with the aim of documenting plants that have potential for production of improved traditional medicines from local resources, as well as plants to be targeted for antimicrobial activity against microorganisms that cause ear, nose and throat infections especially those known to have developed antibiotic resistance.

## Materials and methods

The Central Province of Kenya covers the area around Mount Kenya, where most of the Kikuyus live. The total population is estimated at 3,724,159 inhabitants in an area of 13,191 Km^2^. The Kikuyus are the largest single ethnic group in Kenya and account for 21% of the country's population [27].

**Figure 1:**
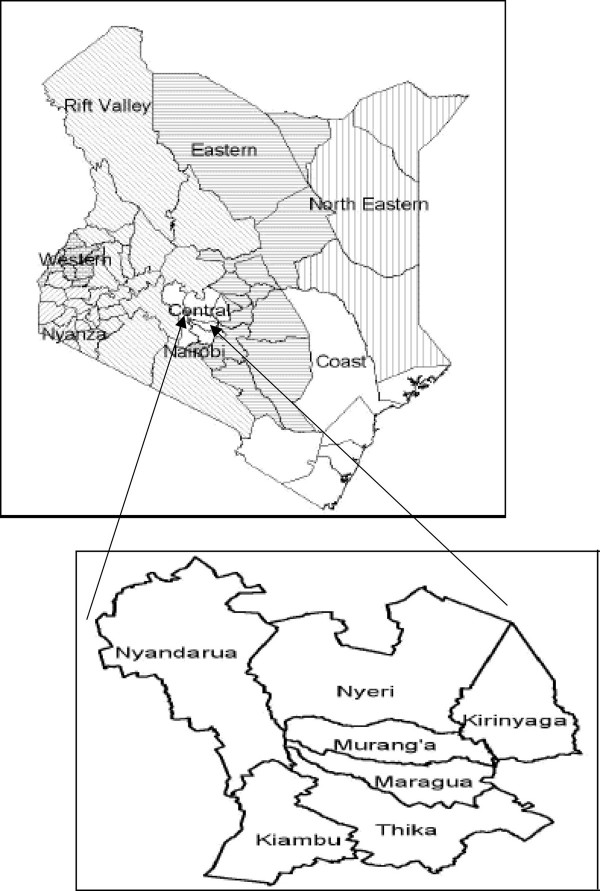
Main provinces in Kenya and constituent districts where fieldwork was done.

This study was part of a larger ethnobotanical survey involving over one hundred respondents, carried out for three years (2001–2004) among the Kikuyus of Central Kenya, comprising seven districts: Thika, Murang'a, Kiambu, Maragwa, Nyandarua, Kirinyaga and Nyeri (Figure 1). This study focuses on results from 64 respondents who used traditional therapies in management of ENT conditions for self medication or in treating others. Initial contacts were made with research assistants in all the districts to explain the aim of the research. These assistants were local people who knew the kikuyu language well, had lived in the area long enough and were familiar with the local people. Prior informed consent was sought with the respondents who were randomly selected in each area.

During the surveys semi-structured questionnaires were used to carry out the interviews, which targeted common ear, nose and throat infections known to the respondents, plant species used, parts used, methods of preparations as well as administration of the drugs. In some cases interviewees were accompanied in their plant collecting duties and observations made of the plants being collected. Where the respondents were uncomfortable with the questionnaires, discussions and informal interviews were employed and in the process information on traditional management of ENT infections obtained. During discussions, information on combination therapy or poly herbal management of ENT conditions were noted and recorded. Any use of none plant remedies for management of ENT infections were recorded.

Plants said to be useful in managing various ENT ailments during the interviews were visually identified in the field by the respondents. Voucher specimens were collected in duplicates using standard taxonomic procedures particularly recording important features for identification in the herbarium. Each specimen included vital parts such as leaves, stems, flowers, and fruits where available. For small herbaceous plants, whole plants were usually collected. For every specimen collected the vernacular names were also recorded. The specimens were dried in the herbarium and then mounted on sheets.

The collected plant material was identified at the Jomo Kenyatta University Herbarium, using the relevant local floras and other taxonomic literature [28-30]. Assistance in identification was sought from an experienced botanist (Mr Simon Mathenge) of the University of Nairobi Herbarium. The collections at Nairobi University Herbarium as well as Jomo Kenyatta University Herbarium were used to make comparisons with the identified specimens. To systematically collect data on management of ENT diseases in this region, questionnaires, semi-structured interviews, informal interviews and discussions with resource people were applied. Interviews were also supplemented by participant observations and consistent field walks to identify the medicinal plants cited and collect ethnotherapeautic specimens.

## Results and discussion

**Figure 2:**
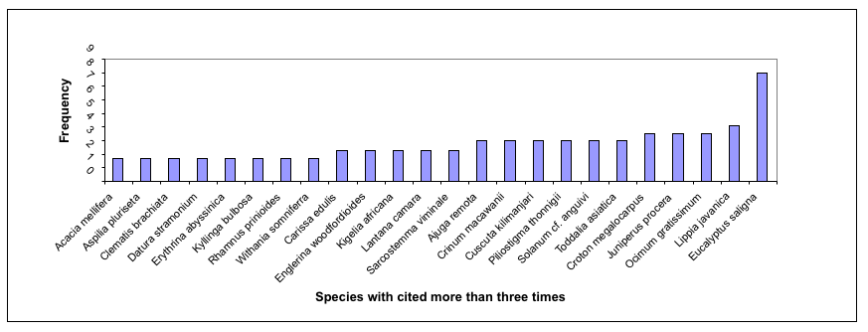
Frequency of plant species used in managing ENT diseases in Central Kenya.

This study found 67 species in 36 plant families as useful in management of ENT conditions in Central Kenya (Table 1) Of these, 24 species were cited three or more times during the field surveys. Some of the species with high frequency of mention included *Eucalyptus saligna*, *Lippia javanica *and *Ocimum gratissimum *(Figure 2). The highest proportion of species used was herbs (37.3%), followed by shrubs (34.3%) and trees (25.4%). Sedges and grasses on the other hand comprised of 3% of the species used in ENT infections management in this region. Whiles one species would have different parts used; this study shows that most of the drug preparations were obtained from leaves (49%) and reasonable amounts from barks (12.5%) and roots (20.5%). Harvesting plant medicines from barks is known to be the most destructive method of harvesting particularly because debarked trees rarely survive [31]. The trees and shrubs from which ENT concoctions are obtained from barks and roots are likely to be in danger of over-exploitation and their conservation status need further investigation.

**Table 1:** Diversity of plant species and methods used in management of ENT diseases in Central Kenya

**Species name**	**Family**	**Kikuyu name**	**Disease**	**Part used**	**Method of drug preparation**	**Method of drug administration**
*Acacia mellifera *(Vahl) Benth.	Mimosaceae	Mûthigira	WC	Bark		Chewing
*Acacia mellifera *(Vahl) Benth.	Mimosaceae	Mûthigira	Cg	Bark	Crushing	Chewing
*Ajuga remota *Benth	Lamiaceae	Wanjirû wa rûriî	CC	Leaves	Boiling	Orally
*Ajuga remota *Benth.	Lamiaceae	Wanjirû wa rûriî	Cg	Leaves	Boiling	Orally
*Asparagus setaceus *(Kunth) Jessop	Asparagaceae	Karûrûra	Cg	Leaves	Crushing/pounding	Chewing
*Aspilia pluriseta *Schweinf.	Asteraceae	Muutî	Cg	Bark	Crushing	Chewing
*Aspilia pluriseta *Schweinf.	Asteraceae	Muutî	OM	Sap	Squeezed from stems	Directly installed
*Bidens pilosa *L.	Asteraceae	Mûcege	NB	Leaves	Boiling	Orally
*Caesalpinia volkensii *Harms	Caesalpiniaceae	Mûbûthî	CC	Roots	Crushing to release sap	Topically by rubbing nasal region
*Carissa edulis *Forssk.	Apocynaceae	Mûkawa	As	Roots Leaves	Boil	Orally
*Carissa edulis *Forssk.	Apocynaceae	Mûkawa	CP	Roots	Boiling Soup added	Orally
*Carissa edulis *Forssk.	Apocynaceae	Mûkawa	CC	Roots	Boiling	Orally
*Citrus aurantiifolia *(Christm.) Swingle	Rutaceae	Ndimu	As	Fruit	Boiling	Orally
*Citrus aurantiifolia *(Christm.) Swingle	Rutaceae	Ndimû	CC	Fruit	Boiling	Orally
*Clematis brachiata *Thunb.	Ranunculaceae	Mûgaya ng'ûndu	CC	Leaves	Boiling to release steam	Inhalation of steam
*Clutia abyssinica *Jaub. & Spach	Euphorbiaceae	Mûthimambûri	To	Leaves	Boiling	Orally
*Coffea arabica *L.	Rubiaceae	Kahûa/Mûhûa	Cg	Root	Boiling	Orally
*Colocasia antiquorum *Schutt	Araceae	Kîrûtû	OM	Sap	Direct	Directly installed
*Combretum collinum *Fres.	Combretaceae	Mûranjîkî	OM	Leaves	Sap extracted	Directly installed
*Crinum macawanii *Bak.	Liliaceae	Gûtûngûrû kîa ngoma	As	Tuber	Boiling	Topically
*Crinum macawanii *Bak.	Liliaceae	Gîtoka	OM	Leaves	Heated, sap squeeze	Directly installed
*Crinum macawanii *Bak.	Liliaceae	Gîtûngûrû kîa ngoma	To	Bulb	Crushing	Massage
*Crotalaria agatiflora *Schweinf.	Papilionaceae	Mûcingiri wa ngambainî	OM	Leaves	Crushing, some water added	Directly installed
*Croton megalocarpus *Hutch.	Euphorbiaceae	Mûkindûri	Cg	Bark	Pounding	Chewing
*Croton megalocarpus *Hutch.	Euphorbiaceae	Mûkindûri	To	Roots	Pounded	Chewing
*Cuscuta kilimanjari *Oliv.	Convolvulaceae	Thîna	OM	Sap	Collected from stems	Directly installed
*Cussonia holstii *Engl.	Araliaceae	Mûruruku	Cg	Bark	Pounding	Chewing
*Dalbergia lactea *Vatke	Papilionaceae	Mwaritha	As	Wet leaves	Crushed to release sap	Topically
*Datura stramonium *L.	Solanaceae	Ndatura	To	Stems	Crushing	Massage
*Dombeya burgessiniae *Gerrard	Sterculiaceae	Mûkeû	Cg	Bark	Pounding	Chewing
*Englerina woodfordioides *(Schweinf.) Balle	Loranthaceae	Kîeha	CC	Whole	Boiling	Orally
*Erythrina abyssinica *DC.	Papilionaceae	Mûhûtî	Cg	Bark	Boiling	Orally
*Erythrina abyssinica *DC.	Papilionaceae	Mûhûtî	NB	Bark	Boiling	Orally
*Eucalyptus saligna *Smith	Myrtaceae	Mûbaû	CC	Leaves	Boiling	Inhalation of steam
*Euphorbia joyae *Bally & S. Carter	Euphorbiaceae	Kariaria	To	Stem	Boiling	Orally
*Juniperus procera *Engl.	Cupressaceae	Mûtarakwa	CC	Bark	Boiling	Orally
*Kigelia africana *(Lam.) Benth.	Bignoniaceae	Mûratina wa thûkûrîi	CC	Rhizome	Boiling	Orally
*Kyllinga bulbosa *P. Beau.	Cyperaceae	Ngothe	OM	Bulb	Bulb crushed to release sap	Directly installed
*Kyllinga bulbosa *P. Beau.	Cyperaceae	Nyeki/Kîgombe	To	Leaves	Crushing	Chewing
*Lactuca inermis *Forssk.	Asteraceae	Mûthûnga	To	Leaves	Dry leaves in shade and soak in hot water	Orally
*Lantana camara *L.	Verbenaceae	Rûîthiki	CC	Leaves	Crushing of wet leaves	Inhalation
*Lantana camara *L.	Verbenaceae	Mûkenia	OM	Leaves	Crushing	Directly installed
*Lippia javanica *(Burm.f.) Spreng.	Verbenaceae	Mûthirîti	CC	Leaves	Crushing, steeped in hot water or milk	Orally
*Mangifera indica *L.	Anacardiaceae	Mwîembe	As	Leaves	Boil	Orally
*Maytenus senegalensis *(Lam.) Exell	Celastraceae	Mûthuthi	To	Sap	Squeezed from stems	Topically
*Melia azadirachta *L.	Meliaceae	Mûarubaine	To	Leaves	Boiling	Orally
*Melia azadirachta *L.	Meliaceae	Mwarumbaine	CC	Leaves	Boiling	Orally
*Momordica foetida *Schumach.	Cucurbitaceae	Karera	To	Leaves	Crushing	Chewing
*Ocimum basilicum *L.	Lamiaceae	Mûtaa	CC	Leaves	Crushed/Ground, steeped in hot water or milk	Orally
*Ocimum gratissimum *L.	Lamiaceae	Mûkandu	OM	Leaves	Crushed	Directly installed
*Ocotea usambarensis *Engl.	Lauraceae	Mûthaitî	CP	Bark	Boiling	Orally
*Ocotea usambarensis *Engl.	Lauraceae	Mûthaitî	Cg	Bark	Boiling	Orally
*Osyris lanceolata *Hocst & Stendel.	Santalaceae	Mûthaithi	N/T	Leaves	Boiling	Orally
*Physalis peruviana *L.	Solanaceae	Mûnathi	As	leaves	Boiling	Orally
*Piliostigma thonnigii *(Schumach.) Milne-Redh.	Papilionaceae	Mûkûra-ûtukû	Cg	Inner bark	Pounding	Chewing
*Piper capense *L.	Piperaceae	Mûrûngû	CC	Leaves	Boiling	Orally
*Pistacia aethiopica *Kokwaro	Anacardiaceae	Mûheheti	CC	Leaves	Boiling	Orally
*Plectranthus comosus *Sims	Lamiaceae	Maigoya	OM	Leaves	Crushing	Directly installed
*Psidium guajava *L.	Myrtaceae	Mûbera	As	Leaves Dry	Boiling	Orally
*Psidium guajava *L.	Myrtaceae	Mûbera	CC	Leaves	Boiling	Orally
*Pterolobium stellatum *(Forssk.) Brenan	Caesalpiniaceae	Mûtandambogo	CC	Roots	Boiling	Orally
*Rhamnus prinoides *L'Herit	Rhamnaceae	Mûkarakinga	CP	Roots	Boiling Soup added	Orally
*Rhamnus prinoides *L'Herit	Rhamnaceae	Mûkarakinga	CC	Leaves	Boiling	Orally
*Rhamnus prinoides *L'Herit	Rhamnaceae	Mûkarakinga	To	Roots/Stem	Boiling	Orally
*Ricinus communis *L.	Euphorbiaceae	Mûbarîki	Cg	Seeds	Grinding the seeds and cooking the fatty part	Orally
*Rubus apetalus *Poir	Rosaceae	Mûtare	To	Fruit and leaves	Boiling	Orally
*Sarcostemma viminale *(L.) R. Br.	Euphorbiaceae	Ndarû	OM	Stem	Heated, Sap squeezed	Directly installed
*Schkuhria pinnata *(Lam.) Thell.	Asteraceae	Gakuinini	CC	Leaves	Boiling	Orally
*Schkuhria pinnata *(Lam.) Thell.	Asteraceae	Gakuinini	Cg	Roots/Leaves	Boiling	Orally
*Senna didymobotrya *(Fresen.) Irwin & Barneby	Caesalpiniaceae	Mwenû	To	Leaves	Boiling	Orally
*Solanum aculeastrum *Dunal	Solanaceae	Gîtûra	To	Root	Boiling	Orally
*Solanum anguivi *Lam.	Solanaceae	Gatongu	OM	Fruits	Juice squeezed	Directly installed
*Sonchus oleraceus *L.	Asteraceae	Mahiû	CP	Roots	Boiling Soup added	Orally
*Sonchus oleraceus *L.	Asteraceae	Mahiû	To	Roots	Boiling	Orally
*Sphilanthes mauritiania *(Pers.) DC.	Asteraceae	Gatharaita	CC	Flowers	Crushing	Chewing
*Sporobolus pyramidalis *P. Beauv.	Poaceae	Kagutu	Cg	Root	Boiling	Orally
*Tagetes minuta *L.	Asteraceae	Mûbangi	As	Leaves	Boiling	Orally
*Tithonia diversifolia *(Hemsl.) A. Gray	Asteraceae	Marûrû	CC	Leaves	Boiling	Orally
*Tithonia diversifolia *(Hemsl.) A. Gray	Asteraceae	Marûrû	Cg	Leaves/Roots	Boiling	Orally
*Toddalia asiatica *(L.) Lam.	Rutaceae	Mûrûrûwe	CC	Roots	Boiling	Orally
*Toddalia asiatica *(L.) Lam.	Rutaceae	Mûrûrûwe	CC	Roots	Boiling	Inhalation
*Toddalia asiatica *(L.) Lam.	Rutaceae	Mûrûrûwe	Cg	Leaves	Boiling	Orally
*Urtica massaica *Mildbr.	Urticaceae	Thabai	CC	Leaves	Boiling	Orally
*Vernonia lasiopus *O.Hoffm.	Asteraceae	Mûchatha	CC	Leaves	Boiling	Orally
*Vernonia lasiopus *O.Hoffm.	Asteraceae	Mûchatha	OM	Flowers	Sap squeezed	Directly installed
*Warburgia ugandensis *Sprague	Canellaceae	Mûthîga	CC	Bark and leaves	Boiling	Orally
*Withania somnifera *(L.) Dunal	Solanaceae	Mûrumbae	As	Roots	Boiling	Orally
*Withania somnifera *(L.) Dunal	Solanaceae	Mûrumbae	CC	Leaves	Boiling	Orally
*Zanthoxyllum usambarensis *(Engl.) Kokwaro	Rutaceae	Mwikunya	CC	Leaves	Boiling	Orally
*Zehneria scabra *(L.f.) Sond.	Cucurbitaceae	Rwegethia	CC	Leaves	Boiling	Orally

Kikuyu names in brackets

As = Asthma (Githûri kinene, Acima, Gîkorora kia ngûkû)

CC = Common cold (Homa, Kihuti)

Cg = Cough (Ruhaya, Gîkorora)

CP = Chest Pains (Githûri)

NB = Nose bleeding

N/T = Neck/throat (Ngingo)

OM = Otitis media (Njika, bûra)

To = Tonsil (Ngaû)

WC = Wooping cough (Gîkoroa thakame)

**Figure 3:**
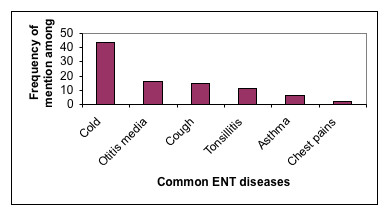
Frequency of ENT diseases managed by traditional herbal preparations.

The most cited ENT disease managed through traditional therapies was found to be the common cold (Figure 3). This condition which has several Kikuyu names (*kǐhuti, homa, njoma*) is managed usually by use of individual species which are mainly boiled and administered by inhalation or leaves crushed and directly inhaled. In a few cases polyherbal preparations were noted especially the mixture of leaves of *Ocimum bacilicum, O. gratissimum *and *lippia javanica *which are boiled together and inhaled while the patient is covered with a thick piece of cloth such as a blanket.

Some studies have shown that viruses are associated with about two-thirds of the cases of common cold among children, while bacteria have been associated with about 4% of the cases. Among these viruses the leading ones are Rhinoviruses which cause common cold in all age groups but enteroviruses are also associated [8]. In the study area, about 30 plant species were found to be utilized for management of common cold. Extracts from these plants need to be a priority in testing for antimicrobial activity for further development of local drugs useful in management of colds.

The current study revealed that 13 plant species were used in managing Otitis media (*Njika/bũra*, in Kikuyu) in Central Kenya (Table 1). Extracts from these plants need to be tested for their activity against some of these bacteria but also against viruses because viruses are known to be a predisposing factor for the development of acute otitis-media [11]. Lubricants from the industries and fat from chicken were also found to be installed into affected ears as a means of managing this condition. Further in some cases the queen stage of the termites were also found to be crushed and the resulting mixture installed in the affected ear(s). In a few cases the tail of the chameleon (alive) is also installed briefly into the affected ear(s). It is not clear whether this has any therapeutic value, but it is worth further investigation.

Otitis-media is known to be the most common bacterial infection especially among children. Sometimes it has severe complications, which have high economic impacts [11]. Studies in Kenya show that this disease causes sensori-neural hearing impairment among pre-school children, in addition to other complications [4]. On a global scale otitis-media is a major indication for antibiotic use with the consequence that some of the microorganisms associated with this condition have acquired antibiotic resistance as in the case of *Pneumoccoci*, *Staphylococcus aureus*, Hemophilus *influenzae *among others [2,10,11]. The traditional therapies cited in this study need to be screened to authenticate their use in managing this condition.

Sixteen plant species were used in managing tonsillitis in Central Kenya. In some of the cases the plant extracts were applied externally as in the case of the sap from *Datura stramonium *stems and bulb of *Crinum macawanii*. These plants are known to have analgesic properties [25] but their antimicrobial activity and those of the species that are either boiled or chewed need to be established. Respiratory viruses such as Adenoviruses, Epstein-Barr viruses, influenza viruses and enteroviruses as well as bacteria are responsible for acute tonsillitis. Some of these for example, hemolytic *Streptococci *have already developed antibiotic resistance [8]. These microorganisms among others need to be the key targets for antimicrobial tests.

Asthma was one of the ENT allergic conditions traditionally managed using plant extracts in Central Kenya. The plants used in this case may be important sources of anti-allergic preparations, which warrant further study.

## Conclusion

This study revealed that the commonest ENT diseases managed through traditional therapies were common cold, Otitis media, cough, tonsillitis, asthma and chest pains. This research further revealed that 67 plant species are used in management of ENT infections in Central Kenya, while 24 of these species are mentioned three or more times during the field survey. There are also some none plant remedies such as fat, oils and animal parts cited in this study.

These remedies form an important database for bioassay guided identification and purification of important therapeutic compounds, antimicrobial trials on their efficacy and further development of improved traditional medicines or other new drugs for management of ENT infections. In the case of polyherbal preparations, their synergistic effects need further investigation. The study also reveals plants whose medicines are obtained in destructive manner and hence these species may need their conservation status to be investigated.

Since ethobotanically-derived phytochemicals have greater activity than compounds derived from random screening and therefore a greater potential for new drug products developed, it is expected that the results of this study will lead to pharmacological investigations with the plants showing reasonable antimicrobial activity.
